# Nitrates of cerium and samarium deposit on human enamel independently of a salivary pellicle

**DOI:** 10.3389/froh.2024.1455924

**Published:** 2024-08-29

**Authors:** Louis Kopp, Karl-Anton Hiller, Fabian Cieplik, Arno Pfitzner, Florian Pielnhofer, Bastian Höfler, Christian Dolle, Áine M. Lennon, Sophia R. Bauer, Wolfgang Buchalla, Konstantin J. Scholz

**Affiliations:** ^1^ Department of Conservative Dentistry and Periodontology, University Hospital Regensburg, Regensburg, Germany; ^2^ Department of Operative Dentistry and Periodontology, Faculty of Medicine, Center for Dental Medicine, Medical Center, University of Freiburg, Freiburg, Germany; ^3^ Institute of Inorganic Chemistry, University of Regensburg, Regensburg, Germany; ^4^ Laboratory for Electron Microscopy, Karlsruhe Institute of Technology, Karlsruhe, Germany; ^5^ Department of Oral and Maxillofacial Surgery, University Hospital Regensburg, Regensburg, Germany

**Keywords:** EDX, SEM, lanthanide, nitrate, caries, cariostatic

## Abstract

**Objectives:**

The aim of this study was to analyze the precipitation of Cerium(III)nitrate hexahydrate [Ce(NO_3_)_3_] or Samarium(III)nitrate hexahydrate [Sm(NO_3_)_3_] solutions on human enamel with and without a salivary pellicle. Investigated parameters were At%Ce and At%Sm measured using energy dispersive x-ray spectroscopy (EDX) after test solution (two concentrations) application.

**Materials and methods:**

Precipitation of Ce(NO_3_)_3_ and Sm(NO_3_)_3_ solutions was examined on human enamel with and without a salivary pellicle. 6 enamel specimens each were obtained from 12 freshly extracted human third molars. These specimens were ground flat and polished. A salivary pellicle was created on 3 of the 6 specimens per tooth by storing the samples in human saliva. Subsequently, an aqueous solution of Ce(NO_3_)_3_ was applied to 2 of the 6 specimens (one with, one without salivary pellicle) for 60 s. The same was carried out with an aqueous solution of Sm(NO_3_)_3_ on 2 further specimens. The remaining 2 specimens from each tooth were treated with demineralized water (negative control). Ce(NO_3_)_3_ and Sm(NO_3_)_3_ solutions were applied at 25 or 50 wt% (aqueous solutions). The test materials and concentrations were distributed using a randomization table. After 60 s exposure and rinsing with demineralized water, the elemental composition (Ce, Sm, Ca, P, O, N, Na, Mg) of the enamel surface was analyzed by EDX. Atomic percentages (At%), differences (ΔAt%) and calcium/phosphorous-ratios (Ca/P-ratios) were calculated and analyzed non-parametrically (*α* = 0.05).

**Results:**

2.0–2.3 At%Ce (median) was detected on Ce(NO_3_)_3_-treated enamel and 0.4–0.7 At% Sm (median) was detected on Sm(NO_3_)_3_-treated enamel. Ce was only detected on the surfaces after application of Ce(NO_3_)_3_, Sm only after application of Sm(NO_3_)_3_. The Ca/P-ratio was significantly lower (1.37–1.59; *p* = 0.028) after the application of 25% and 50%Ce(NO_3_)_3_ as well as 50%Sm(NO_3_)_3_ compared to the control treatment (demineralized water; 1.61–1.63). After treatment with Ce(NO_3_)_3_, At%Ca and At%Na were significantly lower (*p* ≤ 0.043) compared to treatment with Sm(NO_3_)_3_. No significant differences were found between specimens treated with 25% or 50% lanthanide nitrate solution. Presence of a salivary pellicle had no significant influence on the measured At% with the exception of specimens treated with 50% Sm(NO_3_)_3_ with increased At%Sm (*p* ≤ 0.046).

**Conclusions:**

Ce(NO_3_)_3_ and Sm(NO_3_)_3_ precipitate on human enamel independently of the presence of a salivary pellicle.

## Introduction

Dental caries is still the most prevalent non-communicable disease (1), although preventive measures are readily available and widely known in most parts of the world (2). Caries develops as a result of prolonged exposure of the tooth surface to acidic metabolic byproducts generated from bacterial fermentation of dietary carbohydrates (3). This process occurs during phases of demineralization and remineralization of dental hard tissues (4). Demineralization ranges from initial, asymptomatic stages to complete destruction of the tooth (5) and can occur in any age group and in both primary and secondary dentitions (4).

In addition to improvements in oral hygiene and dietary changes, the link between fluoride use and reduced caries prevalence has been known for many years (6). A distinction is made between systemic fluoridation measures, e.g., fluoridated drinking water or table salt which were discovered in the first half of the 20th century and topical measures such as fluoride-containing oral health products, especially toothpastes, introduced in the 1950s (7–9).

Recent studies have shown that the effect of topical fluoride is much greater compared to that of systemic intake during tooth development (10, 11). The topical effects are based on several mechanisms, including accumulation in plaque and on dental hard tissues (12), enhancement of remineralization with Ca^2+^ and PO_4_^3−^ ions from saliva, formation of a surface layer of calcium fluoride (CaF_2_) which dissolves at low pH and thus has a remineralizing effect due to the F^−^ ions released (8, 13).

Meanwhile, numerous other substances with caries-preventive potential have emerged and are the subject of current research (14). Lanthanide salts such as Cerium(III)chloride (CeCl_3_), exhibited anti-erosive and cariostatic effects in *in vitro* studies (15, 16). Caries-like enamel lesions showed reduced lesion progression in demineralization solution after application of CeCl_3_ (16, 17). With respect to erosive dentine lesions, a combined treatment with lanthanide solutions and fluoride resulted in an even higher acid-resistance than fluoride alone (18). Ce was shown to be able to accumulate and remain on bovine enamel surfaces following CeCl_3_ treatment detected by energy dispersive x-ray spectroscopy and scanning electron microscopy (14). In contrast to F^−^ ions, which may replace hydroxide ions in the hydroxyapatite lattice, it is assumed that Ce can substitute Ca in the crystals, resulting in increased acid resistance in hydroxyapatite and enamel (14). Ce and Ca have similar ionic radii in a presumed coordination that would allow Ce^3+^ to replace Ca^2+^ within the hydroxyapatite lattice (19, 20). The substitution of Ca^2+^ with Ce^3+^ could lead to deprotonation of the hydroxyl group within hydroxy apatite (21). This mechanism of substitution of Ca^2+^ with lanthanides (Ca^2+^+H^+^→Ln^3+^) could lead to stabilization of the crystal lattice by eliminating the vibration of the corresponding hydroxide ion (22). Another lanthanide with a similar ionic radius to Ca and Ce is Sm, which for this reason might also be of interest concerning its potential to substitute Ca (19). In contrast to the studies mentioned above, which investigated the effect of CeCl_3_, we decided to analyze the effect of nitrates of the lanthanides Ce and Sm to reduce the influence of the anion as Cl^−^ may substitute OH^−^ within hydroxy apatite (23).

For a substance to have a cariostatic effect, it must be able to interact with the dental hard tissue surface. However, in the clinical situation, the dental surface is typically coated with a layer of salivary proteins, the so-called salivary pellicle (24), hereinafter referred to as pellicle. It consists of proline-rich proteins, statherin, histatins, mucins, amylase, cystatins, lysozyme, and lactoferrin which attach to the tooth surface through their calcium-binding domains (25). The pellicle modulates biological functions between dental hard tissues and the oral environment and it can act as a barrier against acidic substances (26). Consequently, it can be hypothesized that the lanthanides need to penetrate the pellicle to come into contact with the underlying tooth surface or even interact with the pellicle itself.

The null-hypothesis of the present study was that aqueous solutions of Ce(NO_3_)_3_ and Sm(NO_3_)_3_ do not interact with, hence do not influence the elemental composition of enamel surfaces. It was tested by examining enamel specimens treated with different concentrations of lanthanide nitrates in the presence or absence of a salivary pellicle.

## Materials and methods

### Specimen acquisition

Twelve freshly extracted caries-free human third molars (Department of Oral and Maxillofacial Surgery, University Hospital Regensburg) were used for the experiments. These teeth had not been exposed to the oral cavity as ensured by pre-surgical orthopantomogram investigation. After extraction, the teeth were stored for up to 3 months in a solution of 0.5% Chloramine T (Merck KGaA, Darmstadt, Germany; pH 7.1) at 4°C until further processing. The use of extracted human teeth was approved by the ethics committee of the University of Regensburg (ref. 19-1327-101). Each donor gave informed consent. All experiments were performed in accordance with relevant regulations and guidelines.

### Saliva filtration

Saliva was collected from three healthy human donors, purified up to a filtration size of 0.2 µm (Acrodisc Syringe Filters, Pall Medical, Fribourg, Switzerland) according to an established protocol (27) and stored at −80°C until being used to create a pellicle on the specimens. The use of human saliva was approved by the ethics committee of the University of Regensburg (ref. 17-782_2-101). Each donor gave written informed consent.

### Preparation of the test materials

10 ml each of a 25% (w/v) and a 50% (w/v) solution of Ce(NO_3_)_3_ and Sm(NO_3_)_3_ were prepared from 99.9% Ce(NO_3_)_3_ hexahydrate and 99.9% Sm(NO_3_)_3_ hexahydrate (Chempur, Karlsruhe, Germany) and ultrapure water (0.055 µS × cm^−1^, TKA GenPure, TKA xCAD, TKA Wasseraufbereitungssysteme GmbH, Niederelbert, Germany). The test solutions were stored at 4°C and brought to room temperature just before use. The pH-value of the test solutions was checked every two weeks (HI 2211, Hanna Instruments, Vöhringen, Germany) to ensure that the solutions remained stable and the pH-value did not change over the course of the experiments. The demineralized water was produced with the in-house demineralization system (EviroDTS reverse osmosis system, electrical conductivity of 0.34 µS/cm, EnviroDTS GmbH, Friedberg, Germany).

### Preparation of the specimens

**Figure 1:**
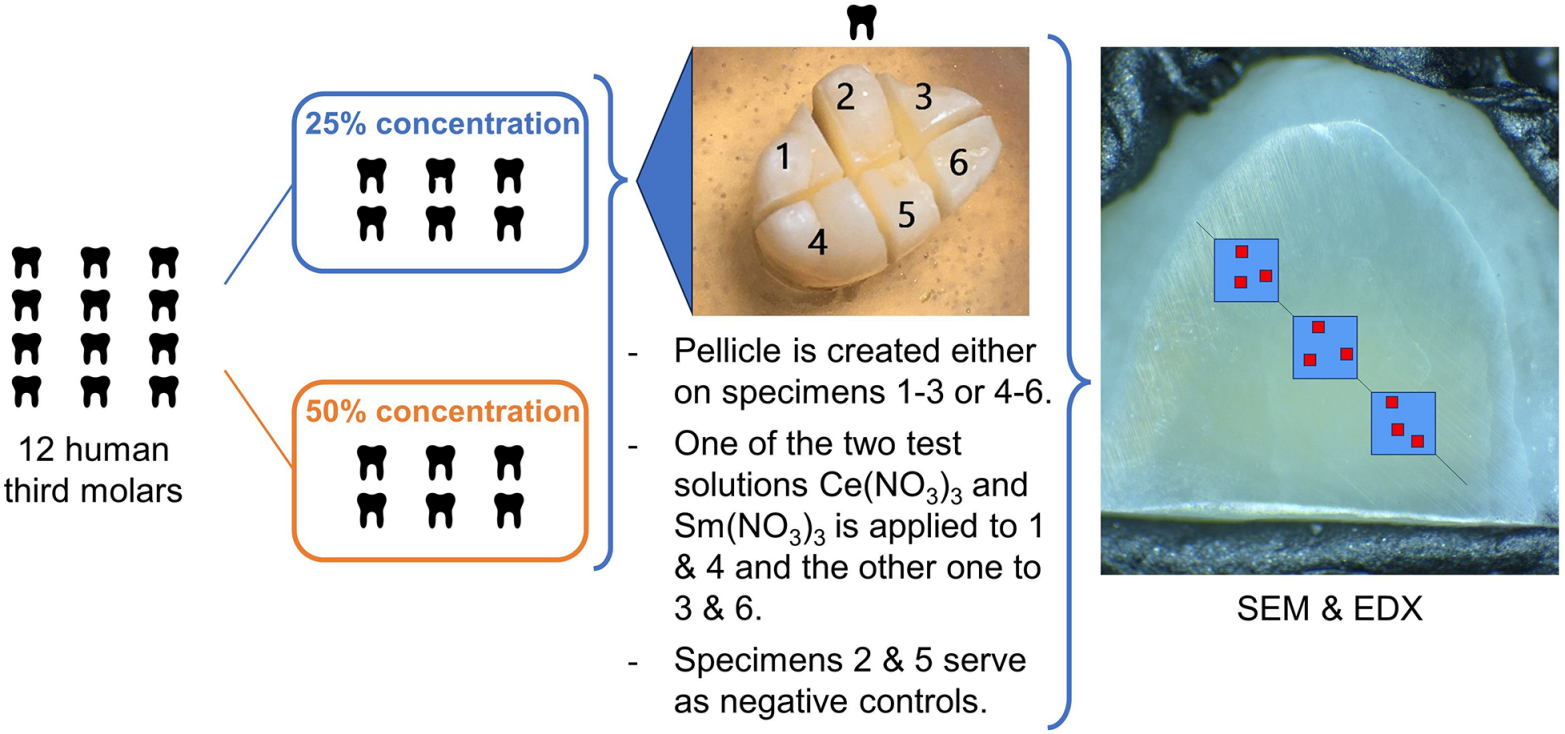
Specimen and treatment allocation. SEM&EDX: Three ROIs visualized by blue boxes, were defined along a virtual line on each specimen. Within these ROIs, the red boxes symbolize the areas where SEM micrographs at an original magnification of ×6,000 and EDX were performed.

Each tooth was placed in demineralized water (osmosis treatment, 0.34 µS × cm^−1^) for 24 h to remove residues of the Chloramine T solution. Then, the teeth were embedded by their roots in a socket made of self-curing dental acrylic (Paladur, Kulzer, Hanau, Germany) for better handling. The crown of each tooth was cut into 6 specimens using a dental handpiece and a water-cooled cutting disc (Superdiaflex H 365F 190 Horico Dental, Berlin, Germany) (Figure 1). Roots and remnants of pulpal tissue were removed from each specimen, and a central enamel surface area of at least 2 × 2 mm was ground flat and polished with SiC abrasive paper under continuous water cooling (Metaserv Motopol 8; 150 rpm, FEPA P1200, CarbiMet, 20 s, FEPA P4000, MicroCut, 20 s; all Buehler, Leinfelden-Echterdingen Germany). Specimens were checked visually using loupes (SV2 2,7× magnification, StarMed GbR, Grafing b. München, Germany) and a microscope (Stereomikroskop WILD M5A, Wild Heerbrugg AG, Switzerland) to ensure all were uniformly polished. The polished enamel surface was cleaned with a polishing fleece (MasterTex Polishing Cloth, Buehler, Germany) and demineralized water. Then, the specimens were treated according to the following scheme (Figure 1): each tooth was allocated to either 25% or 50% concentration of the test solutions via a randomization table. The six specimens from each tooth were numbered. A pellicle was created either on specimens 1–3 or specimens 4–6 according to the randomization table by putting each one in 500 µl of the filtrated saliva solution for 120 min and subsequently rinsing it with demineralized water for 60 s to remove excess saliva. The other half of the specimens remained without pellicle. Without further agitation, 20 µl of each test solution were pipetted onto specimens 1, 3, 4 and 6, as predefined in the randomization table. After 60 s, any residue of the test solutions was removed by rinsing the specimens with 100 ml demineralized water for 30 s. Specimens 2 and 5 served as untreated controls (hereafter called control), one without and one with a pellicle. According to this regimen, test solutions of the same concentration of both Ce(NO_3_)_3_ and Sm(NO_3_)_3_ could be tested on enamel originating from the same tooth both, with and without pellicle. In addition, a control with and without pellicle was also available from each tooth (Figure 1). Each group (Ce(NO_3_)_3_ and Sm(NO_3_)_3_ each in 25% and 50% concentration as well as each with or without pellicle which resulted in 8 groups) was tested on 6 specimens, respectively.

Directly after the application of the lanthanide nitrates, the specimens were mounted onto aluminum stubs (Baltic Präparation e.K., Wetter, Germany) using double-sided adhesive carbon discs and conductive adhesive paste (Leit-C-Tab and Leit-C-Plast, Baltic Präparation e.K., Wetter, Germany) to be examined and analyzed by scanning electron microscopy (FEI Quanta 400 FEG, Thermo Fisher Scientific, FEI Deutschland GmbH, Dreieich, Germany) and EDX without previous sputtering in low vacuum mode (1.5 Torr) (28).

### Surface visualization using backscatter scanning electron microscopy (BSEM)

Three regions of interest (ROIs) were defined on each specimen along an imaginary line from top left to bottom right of the flattened and polished surface (Figure 1). A superficial backscatter scanning electron microscopy (BSEM) micrograph was taken from the enamel surface of each ROI in low vacuum mode (LV) as an overview (backscatter electron mode, 1.5 Torr, accelerating voltage 10 kV, working distance 10 mm, horizontal field width 0.34 mm, original magnification ×800) without previous sputtering, using a gaseous analytical backscatter electron detector (GAD, Thermo Fisher Scientific, FEI Deutschland GmbH, Dreieich, Germany). Within each ROI, another three micrographs were taken at an original magnification of ×6,000 (horizontal field width 45.07 µm, backscatter electron mode, 1.5 Torr, accelerating voltage 10 kV, working distance 10 mm). When microstructural particularities were noticed, they were documented at an original magnification of ×25,000 (horizontal field width 10.82 µm, backscatter electron mode, 1.5 Torr, accelerating voltage 10 kV, working distance 10 mm).

### Determination of surface elemental composition using energy dispersive x-ray spectroscopy (EDX)

EDX was performed on each of the 9 areas per specimen (Figure 1) for measuring the elemental composition of the surface of the enamel (EDAX Octane Elect detector, APEX v2.5 AMETEK EDAX GmbH, Unterschleissheim, Germany). The EDX measurements were performed in low vacuum mode (gaseous analytical detector GAD, 1.5 Torr, accelerating voltage 10 kV, working distance 10 mm, 50 µm aperture, 100 liveseconds, amp time 3.84 µs, resolution 1,024 × 800 pixels). Atomic percent (At%) of the elements Ce, Sm, Ca, P, O, N, Na and Mg were calculated from every area. At%Ce, At%Sm, At%Ca and At%P were the target parameters of the experiment being selective indicators for precipitation of Ce, Sm and the possible substitution of Ca by the applied test solutions.

### Cross sectional scanning transmission electron microscopy analyses (STEM-EDX)

One exemplary specimen each was selected from the groups of specimens treated with 50% Ce(NO_3_)_3%_ and 50% Sm(NO_3_)_3_ without pellicle but with microscopically visible precipitates for examination by transmission electron microscopy (Tecnai Osiris, Thermo Fisher Scientific, FEI Deutschland GmbH, Dreieich, Germany) in cooperation with the KIT (Karlsruhe Institute of Technology, Germany). These specimens were sputtered (platin) and a homogeneous surface area was selected on both specimens using SEM images. A cross section was cut from each selected area, using a focused ion beam (FIB). The cross section was analyzed using high-angle annular dark-field imaging (HAADF-STEM) and EDX. Elements of interest in the EDX were Ce, Sm, Ca and P.

### Data analysis

Within each ROI, the median At% of the three areas was used as descriptive value for the ROI. The median of the three ROI values in turn was used as descriptive value for the specimen. Subsequently, group medians (25%–75% quartiles) for each test material and concentration with and without pellicle were calculated. Differences (ΔAt%) for each element were calculated between each experimental specimen (minuend) with test solution application and the corresponding control specimen (subtrahend) without test solution application from the same tooth.

Data were analyzed statistically by applying non-parametric procedures using SPSS for Windows, v. 29 (SPSS Inc., Chicago, IL, USA). Mann-Whitney *U* tests were used to test for statistically significant differences between independent groups and Wilcoxon-signed-rank tests were used to test for statistically significant differences between dependent groups. The level of significance was set to *α* = 0.05. For evaluation of the influence of the pellicle, the level of significance was adjusted to α*(k) = 1-(1-*α*)^1/k^ with the error rates method (k = number of paired tests to be considered).

## Results

The elemental compositions (At%) and *p*-values of pairwise tests between different parameters are shown in Tables 1–5. Ca, P, O, Na and Mg were detected on the control specimens. The Ca/P-ratio on the controls with pellicle was 1.61 and on the controls without pellicle was 1.63. Neither of the target elements Ce and Sm were detected on the controls. At%Ce >0 was only detected on specimens which had been treated with Ce(NO_3_)_3_ and At%Sm >0 was only detected on specimens that had been treated with Sm(NO_3_)_3_. At%N >0 was not detected on any specimen, regardless of whether a pellicle was created prior to application of the test solution or not. Specimens treated with Ce(NO_3_)_3_ showed median values of 2.0–2.3 At%Ce and those treated with Sm(NO_3_)_3_ showed median values of 0.4–0.7 At%Sm.

**Table 1:** At% (medians, 25%–75% quartiles) of all measured elements at the enamel surface.

	Pellicle	At%Ce			At%Sm			At%Ca			At%P		
		Median	25%	75%	Median	25%	75%	Median	25%	75%	Median	25%	75%
Control	−	0.0	0.0	0.0	0.0	0.0	0.0	20.4	19.5	22.3	12.5	12.2	13.6
	+	0.0	0.0	0.0	0.0	0.0	0.0	19.6	18.8	20.6	12.1	11.8	12.8
25% Ce(NO_3_)_3_	−	2.0	1.4	3.3	0.0	0.0	0.0	17.1	15.1	17.4	12.0	11.9	12.2
	+	2.0	1.4	3.3	0.0	0.0	0.0	16.5	15.1	18.0	12.1	11.5	12.6
50% Ce(NO_3_)_3_	−	2.0	1.6	3.5	0.0	0.0	0.0	17.4	14.4	18.1	12.2	11.8	12.4
	+	2.3	1.7	2.5	0.0	0.0	0.0	17.0	16.5	17.4	12.3	11.9	12.5
25% Sm(NO_3_)_3_	−	0.0	0.0	0.0	0.4	0.3	0.5	20.0	18.8	20.4	12.6	12.0	12.9
	+	0.0	0.0	0.0	0.4	0.2	0.5	19.6	19.2	20.9	12.4	12.2	13.1
50% Sm(NO_3_)_3_	−	0.0	0.0	0.0	0.4	0.2	0.5	19.9	18.8	21.6	12.5	11.9	13.5
	+	0.0	0.0	0.0	0.7	0.4	1.0	20.0	18.5	20.5	12.7	12.4	13.1
	Pellicle	At%O			At%N			At%Na			At%Mg		
		Median	25%	75%	Median	25%	75%	Median	25%	75%	Median	25%	75%
Control	−	66.7	63.5	67.8	0.0	0.0	0.0	0.2	0.2	0.2	0.2	0.2	0.2
	+	67.7	66.1	69.1	0.0	0.0	0.2	0.2	0.2	0.2	0.2	0.2	0.2
25% Ce(NO_3_)_3_	−	68.8	67.9	69.3	0.0	0.0	0.0	0.1	0.1	0.1	0.2	0.2	0.2
	+	68.8	67.0	70.3	0.0	0.0	0.2	0.1	0.1	0.1	0.2	0.2	0.2
50% Ce(NO_3_)_3_	−	68.3	67.4	69.6	0.0	0.0	0.0	0.1	0.1	0.1	0.2	0.2	0.2
	+	68.0	67.4	69.2	0.0	0.0	0.1	0.1	0.1	0.1	0.2	0.2	0.2
25% Sm(NO_3_)_3_	−	66.6	65.8	68.0	0.0	0.0	0.0	0.2	0.2	0.2	0.2	0.2	0.2
	+	67.1	64.9	67.9	0.0	0.0	0.0	0.2	0.2	0.2	0.2	0.2	0.2
50% Sm(NO_3_)_3_	−	67.0	64.0	68.5	0.0	0.0	0.0	0.2	0.2	0.2	0.2	0.2	0.2
	+	66.4	65.5	67.6	0.0	0.0	0.0	0.2	0.2	0.2	0.2	0.2	0.2

Presentation of the medians and quartiles, *n* = 12.−without pellicle,+with pellicle.

**Table 2:** *P*-values from pairwise tests of all measured elements (At%) on the specimens without and with pellicle treated with 25% or 50% Sm(NO_3_)_3_ or Ce(NO_3_)_3_ vs. the control-specimens and specimens treated with 25% Sm(NO_3_)_3_ and Ce(NO_3_)_3_ vs. specimens treated with 50% concentration of Sm(NO_3_)_3_ and Ce(NO_3_)_3_.

		Without pellicle			With pellicle		
		Control vs. 25%	Control vs. 50%	25% vs. 50%	Control vs. 25%	Control vs. 50%	25% vs. 50%
Ce(NO_3_)_3_	Ce	**0.028**	**0.028**	n.s.	**0.028**	**0.027**	n.s.
	Sm	n.s.	n.s.	n.s.	n.s.	n.s.	n.s.
	Ca	**0.028**	**0.028**	n.s.	**0.028**	**0.028**	n.s.
	P	n.s.	n.s.	n.s.	n.s.	n.s.	n.s.
	O	n.s.	n.s.	n.s.	n.s.	n.s.	n.s.
	N	n.s.	n.s.	n.s.	n.s.	n.s.	n.s.
	Na	**0.046**	**0.014**	n.s.	**0.014**	**0.014**	n.s.
	Mg	n.s.	n.s.	n.s.	n.s.	n.s.	n.s.
Sm(NO_3_)_3_	Ce	n.s.	n.s.	n.s.	n.s.	n.s.	n.s.
	Sm	**0.027**	**0.042**	n.s.	**0.027**	**0.028**	n.s.
	Ca	n.s.	n.s.	n.s.	n.s.	n.s.	n.s.
	P	n.s.	n.s.	n.s.	**0.042**	n.s.	n.s.
	O	n.s.	n.s.	n.s.	**0.028**	n.s.	n.s.
	N	n.s.	n.s.	n.s.	n.s.	n.s.	n.s.
	Na	n.s.	n.s.	n.s.	n.s.	n.s.	n.s.
	Mg	n.s.	n.s.	n.s.	n.s.	n.s.	n.s.

n.s., not statistically different (*p* > 0.05).

Bold *p*-values: statistically significant difference.

**Table 3:** Pairwise comparisons of all ΔAt% for the specimens without and with pellicle treated with 25% or 50% Sm(NO_3_)_3_ vs. all ΔAt% for the specimens without and with pellicle treated with 25% or 50% Ce(NO_3_)_3_.

	Ce(NO_3_)_3_ vs. Sm(NO_3_)_3_			
	Without pellicle		With pellicle	
	25% concentration	50% concentration	25% concentration	50% concentration
Ce	**0.028**	**0.028**	**0.028**	**0**.**027**
Sm	**0.027**	**0.042**	**0.027**	**0**.**028**
Ca	**0.028**	**0.043**	**0.028**	**0**.**028**
P	n.s.	n.s.	n.s.	**0**.**042**
O	**0.028**	n.s.	n.s.	**0**.**028**
N	n.s.	n.s.	n.s.	n.s.
Na	**0.025**	**0.014**	**0.014**	**0**.**014**
Mg	n.s.	n.s.	n.s.	n.s.

n.s., not statistically different (*p* > 0.05).

Bold *p*-values: statistically significant difference.

**Table 4:** Pairwise comparisons of all all ΔAt% for specimens treated with 25% or 50% Sm(NO_3_)_3_ and 25% or 50% Ce(NO_3_)_3_ depending on the presence of a pellicle.

	Without pellicle vs. with pellicle			
	Ce(NO_3_)_3_		Sm(NO_3_)_3_	
	25% concentration	50% concentration	25% concentration	50% concentration
Ce	n.s.	n.s.	n.s.	n.s.
Sm	n.s.	n.s.	n.s.	**0.046**
Ca	n.s.	n.s.	n.s.	n.s.
P	n.s.	n.s.	n.s.	n.s.
O	n.s.	n.s.	n.s.	n.s.
N	n.s.	n.s.	n.s.	n.s.
Na	n.s.	n.s.	n.s.	n.s.
Mg	n.s.	n.s.	n.s.	n.s.

n.s., not statistically different (*p* > 0.05).

Bold *p*-values: statistically significant difference.

**Table 5:** *P*-values for pairwise comparisons of the Ca/P-ratios on all specimens.

	Control	25% Ce(NO_3_)_3_	50% Ce(NO_3_)_3_	25% Sm(NO_3_)_3_	50% Sm(NO_3_)_3_	
Control	n.s.	**0.028**	**0.028**	n.s.	**0.028**	Without pellicle
25% Ce(NO_3_)_3_	**0.028**	n.s.	n.s.	**0.028**	n.t.	
50% Ce(NO_3_)_3_	**0.028**	n.s.	n.s.	n.t.	**0.028**	
25% Sm(NO_3_)_3_	n.s.	**0.028**	n.t.	n.s.	n.s.	
50% Sm(NO_3_)_3_	**0.028**	n.t.	**0.028**	n.s.	**0.028**	
	With pellicle					With vs. without pellicle

n.t., not tested; n.s., not statistically different (*p* > 0.05).

Bold *p*-values: statistically significant difference.

**Figure 2:**
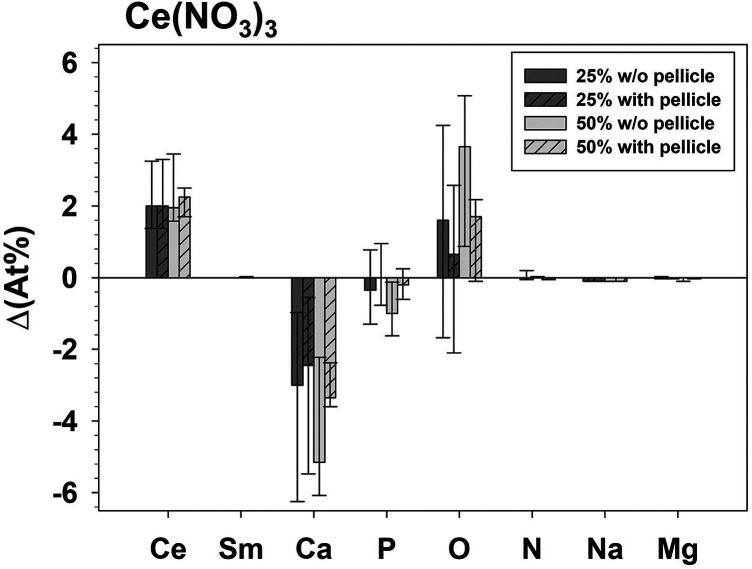
Δ(At%) - difference of the analyzed elements between specimens treated with Ce(NO_3_)_3_ (minuend) compared to the respective untreated control specimens (subtrahend).

**Figure 3:**
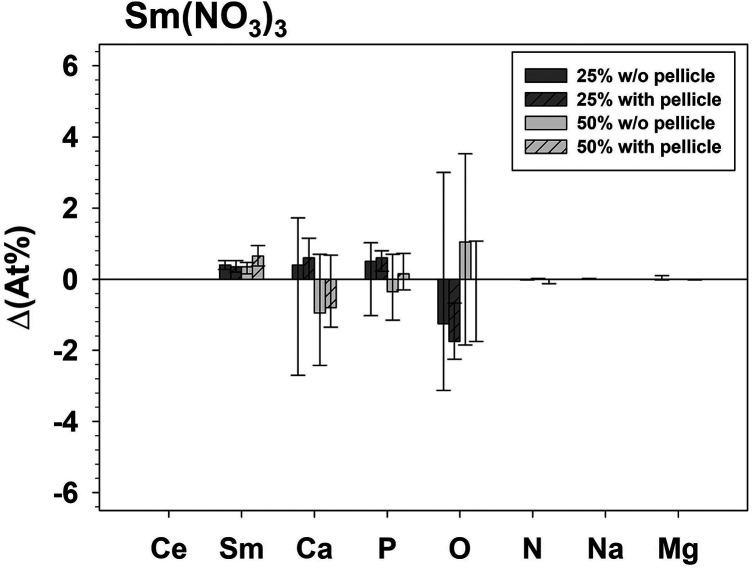
Δ(At%) - difference of the analyzed elements between specimens treated with Sm(NO_3_)_3_ (minuend) compared to the respective untreated control specimens (subtrahend).

The influence of test solution application is shown in the following figures (Figures 2, 3) by the difference (ΔAt%) between the samples treated with test solution and their corresponding controls.

**Figure 4:**
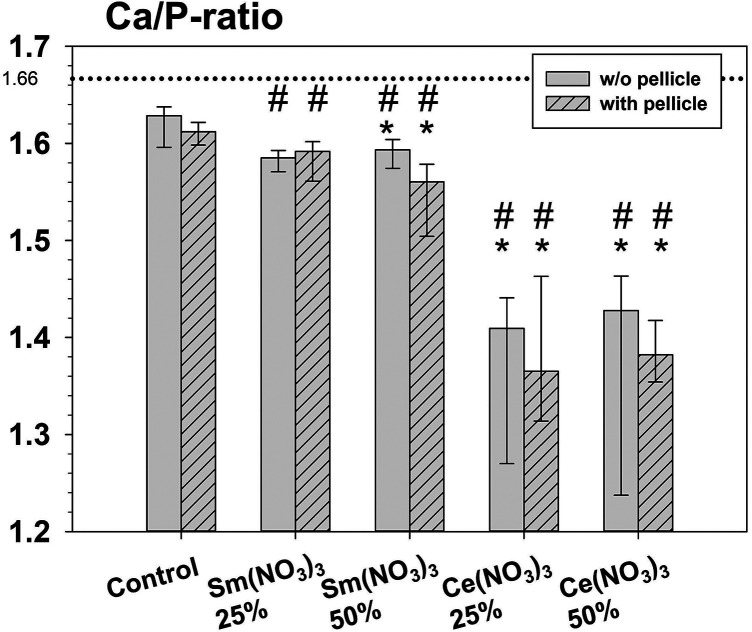
Calculated Ca/P-ratios. Asterisks (*) indicate significant differences between specimens treated with a test solution and the respective control. Hashtags (#) indicate significant differences between Ce(NO_3_)_3_ and Sm(NO_3_)_3_ under respective conditions (with or without pellicle).

The presence of a pellicle had a significant influence (*p* = 0.046) exhibiting a higher ΔAt%Sm only for the group treated with 50% Sm(NO_3_)_3_. The influence of the test solution Ce(NO_3_)_3_ or Sm(NO_3_)_3_ on ΔAt% was significant for Ce (*p* ≤ 0.028), Sm (*p* ≤ 0.042), Ca (*p* ≤ 0.043), and Na (*p* ≤ 0.025) for both concentrations with and without pellicle. ΔAt%O differed significantly between application of Ce(NO_3_)_3_ or Sm(NO_3_)_3_ for 25% (*p* = 0.028) without pellicle and 50%. The concentration (25% or 50%) did not significantly influence the ΔAt% of any element for application of Ce(NO_3_)_3_ or Sm(NO_3_)_3_.

All specimens treated with 25% or 50% Ce(NO_3_)_3_ showed a significantly lower Ca/P-ratio compared to untreated controls, regardless of pellicle (Figure 4). The same was found for the specimens treated with 50% Sm(NO_3_)_3_. Only the Ca/P-ratio of the specimens treated with 25% Sm(NO_3_)_3_ did not vary significantly compared to the controls. The pellicle itself led to a lower Ca/P-ratio only on the specimens treated with 50% Sm(NO_3_)_3_, otherwise it had no significant effect on the Ca/P-ratio.

**Figure 5:**
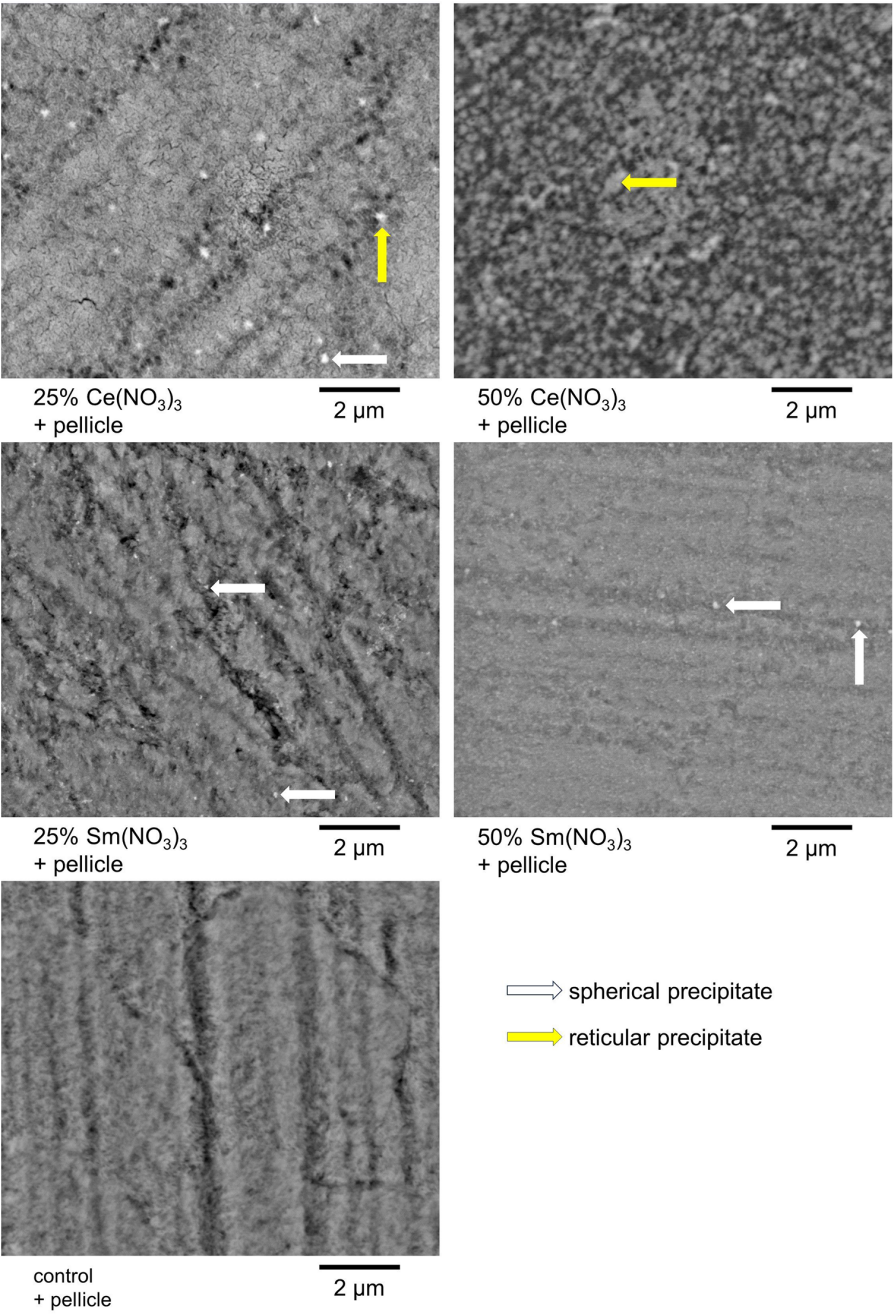
Exemplary BSEM-images of a characteristic form of precipitate for each applied test solution with artificial pellicle.

**Figure 6:**
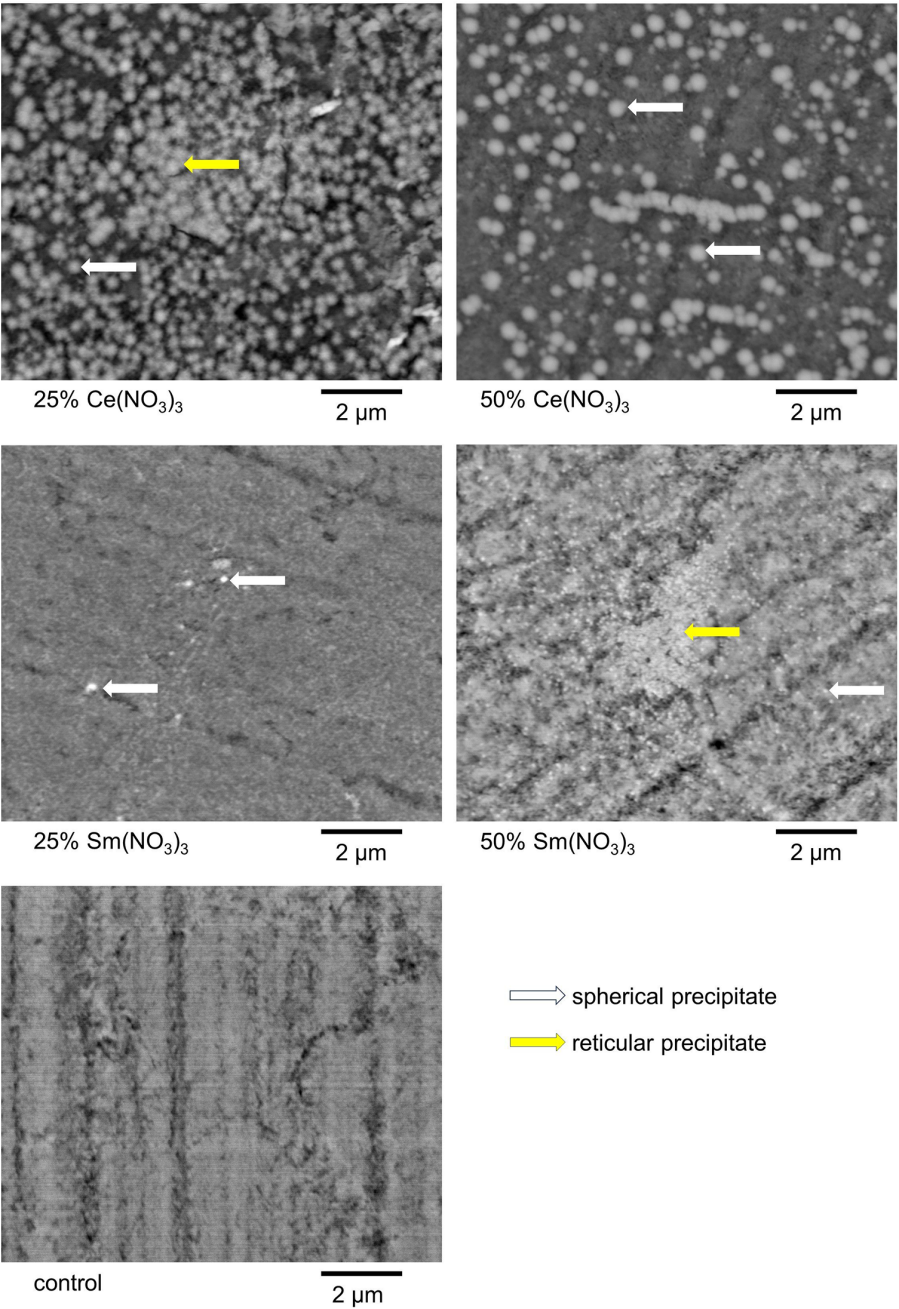
Exemplary BSEM-images of a characteristic form of precipitate for each applied test solution without artificial pellicle. Intriguingly, 25% Ce(NO_3_)_3_ led to denser and more homogeneous precipitates, but 50% Ce(NO_3_)_3_ to bigger precipitates in these examples.

The precipitates that formed on the enamel surface were visualized for all test solutions via BSEM. For each test solution, with and without pellicle, characteristic precipitates are shown in Figures 5, 6. For 25% and 50% Ce(NO_3_)_3_ with pellicle, a combination of reticular and spherical precipitates was visible in most specimens. For 25% Sm(NO_3_)_3_ without pellicle there were hardly any precipitates visible with the exception of spherical precipitates on some specimens. For all other test solutions with or without pellicle, spherical precipitates were seen almost exclusively.

**Figure 7:**
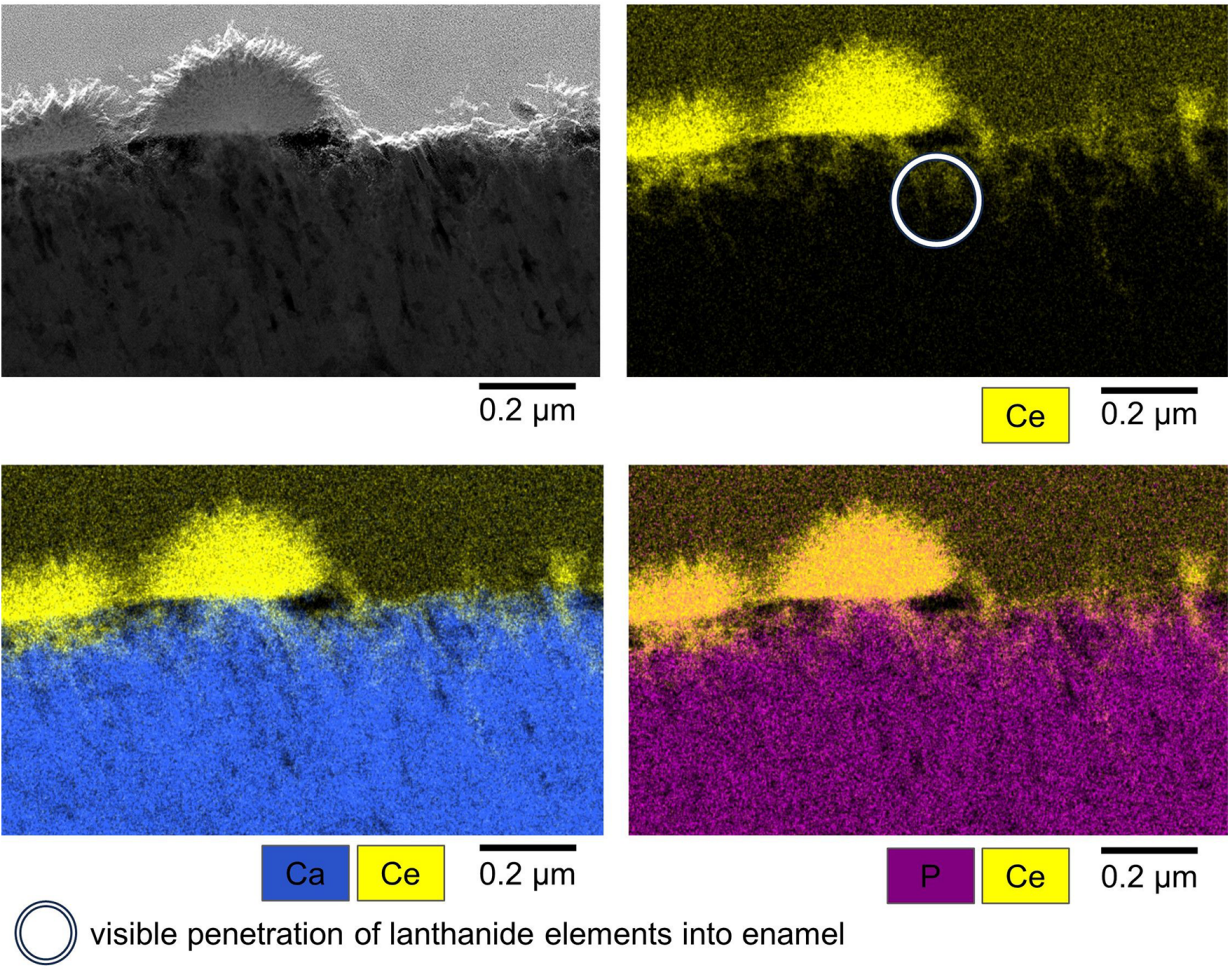
Exemplary STEM image (top left) and EDX mappings of Ce, Ca and P (top right and bottom right and left) of characteristic Ce(NO_3_)_3_ precipitates. White circle: visible penetration of lanthanide elements into enamel.

**Figure 8:**
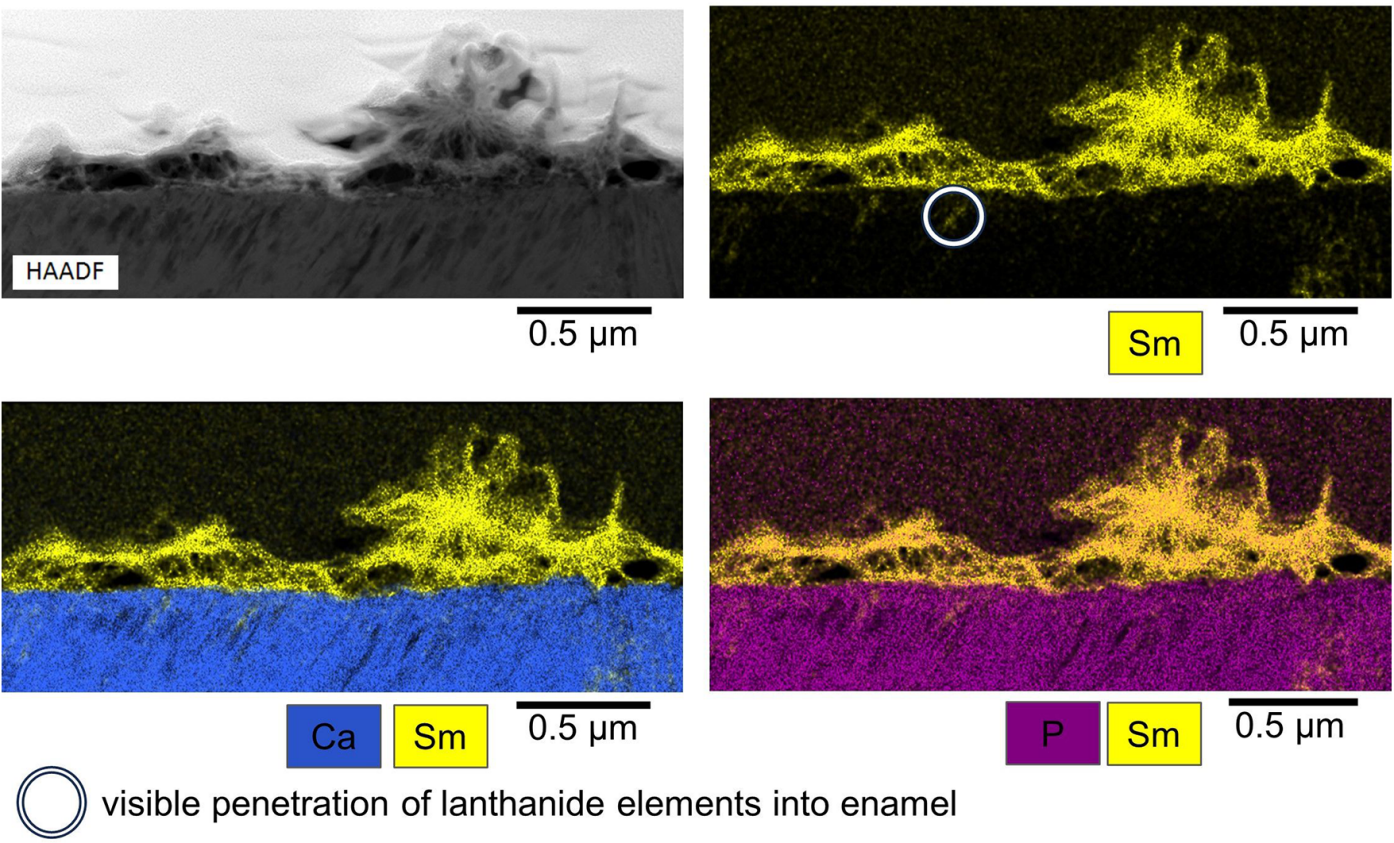
Exemplary STEM image (top left) and EDX mappings of Sm, Ca and P (top right and bottom right and left) of characteristic Sm(NO_3_)_3_ precipitates. White circle: visible penetration of lanthanide elements into enamel.

Both specimens analyzed by cross sectional STEM and EDX showed superficial layers of precipitation consisting of Ce and Sm crystallites with P also present. In addition, both Ce and Sm were detected in deeper layers of the specimens as shown in Figures 7, 8. The pH of the solutions remained constant at acidic values over the course of the experiment, pH: 3.22–3.25 for 25% Sm(NO_3_)_3_, 2.15–2.22 for 50% Sm(NO_3_)_3_, 2.28–2.34 for 25% Ce(NO_3_)_3_ and 1.40–1.45 for 50% Ce(NO_3_)_3_.

## Discussion

### Discussion of the method

Under clinical conditions in the oral cavity, saliva proteins adsorb onto the bare tooth surface immediately and form a pellicle that covers all dental hard tissue surfaces (29). A cariostatic agent must therefore be able to penetrate the pellicle effectively to interact with the tooth structure below and take effect. Half of the specimens were covered with a pellicle by storing them within filtered human saliva to mimic the clinical situation and to evaluate its influence on the interaction between the lanthanide nitrates and the enamel in comparison to specimens without a pellicle. The saliva we used originated from healthy human donors and was filtered up to 0.2 µm to ensure the absence of any bacteria without overly removing saliva proteins (30).

As test solutions, we decided to use solutions of 25 wt% and 50 wt% of Ce(NO_3_)_3_ and Sm(NO_3_)_3_. The higher concentration, 50 wt%, lies near to the maximum solubility of both substances (31, 32) and 25 wt% seemed to be a reasonable gradation to investigate dose-related effects. In the literature, experiments have been conducted with 10 wt% (16, 18), 25 wt% (14) and 50 wt% (33) solutions of lanthanide salts. Ce has already shown its caries-protective and cariostatic potential in several *in vitro* studies with human and bovine dental hard tissues (14, 16–18). Sm was the second lanthanide we chose to investigate, as its ionic radius lies near to that of Ce and Ca what could be an indication of its capability to interact with dental hard tissue in a similar way. In the context of dentistry, Sm is found as an alloy constituent in attachment magnets for dental prostheses until now (34). In earlier studies, the effect of lanthanide salts with dental hard tissues was analyzed using chloride salts (14, 16, 18). Instead of chloride salts, we decided to use nitrates of Ce and Sm to reduce the influence of the anion as Cl^−^ may substitute OH^−^ within hydroxy apatite (23). Furthermore, *in vitro* studies aiming for synthesis of doped hydroxyapatite utilized nitrates of lanthanides. Therefore, an incorporation of lanthanide nitrates into the crystal lattice of hydroxyapatite in human enamel might also be possible (35, 36). Moreover, lanthanide nitrates are highly soluble in water (31, 32) and well available. Other nitrate salts like potassium nitrate have already been added into toothpastes and are approved by the FDA as an ingredient in the treatment against dentine hypersensitivity (37). In another *in vitro* study, Ce(NO_3_)_3_ and Sm(NO_3_)_3_ application led to accumulation and interaction with dentin with and without smear layer, in particular in the smear layer and in the dentin tubules (33). Concerning the toxicology of the test substances, the oral LD50 of Ce(NO_3_)_3_ (4,200 mg/kg body weight, rat) and Sm(NO_3_)_3_ (2,900 mg/kg body weight, rat) (38) is higher than the human LD50 of sodium chloride (2,690 mg/kg body weight) (39). A study evaluating the subchronic toxicity of Ce(NO_3_)_3_ in Wistar rats found a no observed adverse effect level (NOAEL) of 75 mg/kg body weight, which is 30 times higher than the lowest reported NOAEL from a chronic animal toxicity study that investigated systemic effects of fluoride (40).

To show the precipitation of the target substances on the specimens, the SEM micrographs and EDX were taken in low vacuum mode and low voltage with a magnification of ×800, ×6,000 and ×25,000. This method allows a high material-contrast, as a sputter-coating to increase the conductibility of the specimen surface is not needed (14, 41). We decided to perform the EDX measurements by setting ROIs analogously to a previous study where fluoride preparations were applied to enamel in the same mode as the test materials in the present study (14).

In addition to the SEM and EDX of the specimen surfaces, exemplary STEM micrographs and STEM-EDX were performed to analyze the precipitates in more detail and to evaluate whether cerium and samarium can penetrate into deeper layers of the enamel specimens. For this purpose, we selected a specimen that exhibited homogeneous precipitation on the specimen surface as well as both spherical and reticular precipitates in the initial EDX and SEM analysis.

### Discussion of the results

Ca, P, O, Na and Mg were detected on all specimens. In a study that focused on the elemental composition of enamel (42), Ca, P, O and Na was also found on all specimens but Mg was only found on some. Mg, present in the core of enamel crystals (43), is known to create special irregularities in enamel crystals, which lead to higher acid solubility compared to chemically pure hydroxyapatite, in a similar way to the incorporation of carbonate (44). Following application of Ce(NO_3_)_3_, a median of 2.0–2.3 At%Ce indicates that Ce- containing precipitates were formed on the enamel surface. This is in accordance with an earlier study where the application of a 25% solution of CeCl_3_ also led to precipitation of Ce on bovine enamel (14). Likewise, a median of 0.4–0.7 At%Sm indicates formation of Sm containing precipitates. A study investigating the incorporation of various lanthanides into enamel proved that treatment with SmCl_3_ caused the formation of a Sm containing phosphate-complex layer on the enamel surface (45) which supports our findings of Sm precipitation.

Due to our study design, which allowed the calculation of Δ(At%) of the analyzed elements as the difference for the specimens treated with Ce(NO_3_)_3_ or Sm(NO_3_)_3_ (minuend) compared to their respective control specimens (subtrahend), changes in elemental composition caused by lanthanide nitrate treatment could be detected and analyzed in dependent specimens.

Following application of Ce(NO_3_)_3_, At%Ce increased and at the same time At%Ca and At%Na decreased, indicating changes in mineral composition beyond the formation of precipitates. Application of Sm(NO_3_)_3_-solutions did not influence either At%Ca or At%Na. In another study it was found that treatment of enamel with NaF led to a decrease in At%O and At%P on the specimen surface while At%Ca and in At%Na increased (14). Since in the present study At%Ca and At%Na decreased after treatment with Ce(NO_3_)_3_, these elements were possibly substituted within the surface layer. At%N could not be detected on any of our specimens. This contrasts with a study using another methodology, where it was found that *in vivo* and *in vitro* pellicles contain nitrogen (46). Those pellicles were formed on etched enamel and by saliva from only one donor, which was passed through a 0.45 µm filter instead of the 0.2 µm filter used here. More importantly, the pellicle had been removed from the enamel specimens with hydrochloric acid and the nitrogen content of the pellicle was determined by Nessler's reaction which may be more sensitive compared to the EDX-setup of the present study.

The Ca/P-ratio of the enamel surface was influenced by treatment with lanthanide nitrates. The fact that all specimens treated with Ce(NO_3_)_3_ and also those treated with 50% Sm(NO_3_)_3_ showed significantly decreased Ca/P-ratios is in line with the findings of a previous study where an application of CeCl_3_ also led to a lowered Ca/P-ratio (14). There, it was discussed that Ce may act as a substitute for Ca within the hydroxyapatite lattice. This could also apply for Sm, which has a similar ionic radius to Ce and Ca (19) and thus could also substitute Ca when applied onto enamel. The fact that the treatment with 25% Sm(NO_3_)_3_ did not cause a significant change in Ca/P-ratio could be indicative of a less effective interaction of Sm(NO_3_)_3_ with human enamel compared to Ce(NO_3_)_3_. This could be explained by the higher pH-value of the 25% solution, which also results in a less powerful interaction with healthy enamel in the case of fluorides (27).

When the specimens treated with 25% and 50% lanthanide nitrate solutions were directly compared, we could not find any significant difference in the measured At%. In contrast to this, a study focusing on fluorides found that the fluoride uptake in enamel increased with an increasing fluoride concentration (47). We suspect that the same applies to lanthanide nitrates but both the 25% and the 50% concentration may be high enough to saturate the enamel surface so that no difference in precipitation between these concentrations was visible.

Concerning the influence of the pellicle, the only difference observed was an increased At%Sm and decreased Ca/P-ratio on the specimens treated with 50% Sm(NO_3_)_3_. Conversely, it can be concluded that the pellicle has no significant influence on most values. This contrasts with the behaviour of NaF, where At%F was decreased significantly in the presence of pellicle (27). This suggests that the way lanthanide salts interact with human enamel is influenced less by the pellicle than is typically observed for fluorides.

The exemplary cross sectional EDX-mappings based on the TEM-images show that those precipitates consist mainly of Ce and Sm and that they are closely bound to the enamel surface. Phosphate was also detected within the precipitates. However, further studies are necessary to analyze the composition of the precipitates on the enamel surface, e.g., if they contain lanthanide oxides, phosphates or apatite. Beyond that, future studies could focus on the influence that the pH of lanthanide solutions has on their precipitation behavior and their interaction with dental hard tissues.

The exemplary cross sectional EDX-mappings detected Ce and Sm in depths of about 0.2–0.5 µm below the specimen surface, which proves that the lanthanides can penetrate *into* the enamel. Beyond the formation of a lanthanide-rich layer on the enamel surface, this penetration *into* enamel is an important precondition of lanthanides being able to inhibit demineralization and increase remineralization.

The present study showed for the first time a precipitation and interaction of Ce(NO_3_)_3_ and Sm(NO_3_)_3_ when applied to enamel after a short exposure time and despite subsequent rinsing. Limiting factors of the study are the absence of a demineralization- remineralization cycle or an erosion model. Future studies should also investigate the stability of these precipitates when exposed to acid or base, brush abrasion or mastication, as well as repeated application of the substances, different pH-values and incorporation into *in situ* models and clinical studies.

## Conclusion

Within the limitations of this *in vitro* study we can conclude that solutions of Ce(NO_3_)_3_ and Sm(NO_3_)_3_ applied on polished enamel result in superficial accumulation of Ce and Sm as well as penetration into the enamel surface up to 0.5 µm and a change in enamel elemental composition irrespective of the presence of a pellicle.

## Data Availability

All data used in this publication are available within this article. No additional data repositories are required for access.
